# Optimizing Village-Level Targeting of Active Case Detection to Support Visceral Leishmaniasis Elimination in India

**DOI:** 10.3389/fcimb.2021.648847

**Published:** 2021-03-24

**Authors:** Joy Bindroo, Khushbu Priyamvada, Lloyd A. C. Chapman, Tanmay Mahapatra, Bikas Sinha, Indranath Banerjee, Prabhas Kumar Mishra, Basab Rooj, Kumar Kundan, Nupur Roy, Naresh Kumar Gill, Allen Hightower, Madan Prasad Sharma, Neeraj Dhingra, Caryn Bern, Sridhar Srikantiah

**Affiliations:** ^1^ Bihar Technical Support Program, CARE-India Solutions for Sustainable Development, Patna, India; ^2^ Department of Medicine, University of California San Francisco, San Francisco, CA, United States; ^3^ Centre for Mathematical Modelling of Infectious Disease, London School of Hygiene and Tropical Medicine, London, United Kingdom; ^4^ National Vector-Borne Disease Control Programme, Ministry of Health & Family Welfare, Government of India, New Delhi, India; ^5^ Independent consultant, Bangkok, Thailand; ^6^ Department of Health, Government of Bihar, Patna, India; ^7^ Department of Epidemiology and Biostatistics, University of California San Francisco, San Francisco CA, United States

**Keywords:** visceral leishmaniasis, surveillance, epidemiology, India, disease control

## Abstract

**Background:**

India has made major progress in improving control of visceral leishmaniasis (VL) in recent years, in part through shortening the time infectious patients remain untreated. Active case detection decreases the time from VL onset to diagnosis and treatment, but requires substantial human resources. Targeting approaches are therefore essential to feasibility.

**Methods:**

We analyzed data from the Kala-azar Management Information System (KAMIS), using village-level VL cases over specific time intervals to predict risk in subsequent years. We also graphed the time between cases in villages and examined how these patterns track with village-level risk of additional cases across the range of cumulative village case-loads. Finally, we assessed the trade-off between ACD effort and yield.

**Results:**

In 2013, only 9.3% of all villages reported VL cases; this proportion shrank to 3.9% in 2019. Newly affected villages as a percentage of all affected villages decreased from 54.3% in 2014 to 23.5% in 2019, as more surveillance data accumulated and overall VL incidence declined. The risk of additional cases in a village increased with increasing cumulative incidence, reaching approximately 90% in villages with 12 cases and 100% in villages with 45 cases, but the vast majority of villages had small cumulative case numbers. The time-to-next-case decreased with increasing case-load. Using a 3-year window (2016–2018), a threshold of seven VL cases at the village level selects 329 villages and yields 23% of cases reported in 2019, while a threshold of three cases selects 1,241 villages and yields 46% of cases reported in 2019. Using a 6-year window increases both effort and yield.

**Conclusion:**

Decisions on targeting must consider the trade-off between number of villages targeted and yield and will depend upon the operational efficiencies of existing programs and the feasibility of specific ACD approaches. The maintenance of a sensitive, comprehensive VL surveillance system will be crucial to preventing future VL resurgence.

## Introduction

India has made major strides in the control of visceral leishmaniasis (VL, also known as kala-azar) over the past 8 years. Annual incidence has fallen from over 20,000 cases in 2012 to 3,143 in 2019, the lowest level in six decades (National Vector Borne Disease Control Programme, 2020a). As of 2019, 596 of 633 endemic blocks reported case-loads below the kala-azar elimination program target of one case per 10,000 population per year. When CARE India began providing support to the national VL program in 2013, research publications estimated that reported VL incidence represented a four- to eight-fold underestimate (Singh et al., 2006; Singh et al., 2010). In response, assessments of VL reporting completeness were conducted, first in eight districts of Bihar in 2013 (Das et al., 2016), then in the 33 affected districts of Bihar and four affected districts of Jharkhand in 2015. These assessments revealed that approximately 85% of VL cases were eventually detected by passive surveillance (Dubey et al., in press).

These assessments shaped the design and implementation of the Kala Azar Management Information System (KAMIS), the electronic surveillance system used by the national kala-azar elimination program. Bihar and Jharkhand reached virtually universal implementation of KAMIS in 2017, and recent cases are listed in close to real-time. All known, traceable cases since 2013 are listed in KAMIS. West Bengal reached universal usage of KAMIS by 2018 and Uttar Pradesh in 2019. These four states are the only states in which kala-azar has been endemic for well over twenty years.

The 2013 and 2015 situation assessments (Dubey et al., in press) also provided the foundation for active case detection (ACD) methods that have been utilized by CARE field teams since 2017 and are mentioned in the recently issued national guidelines for active case detection as index case-based approaches (National Vector Borne Disease Control Programme, 2020b). A recent analysis showed that around 40% of VL cases are detected by these relatively light-touch methods, which currently target villages with at least one case in the previous year, and that ACD is associated with a significantly shorter time to diagnosis compared to passive case detection during the same time period (Dubey et al., in press).

The well-documented clustering of VL cases at the village and sub-village levels provides the basis for developing ACD targeting strategies (Bulstra et al., 2018; Chapman et al., 2020; Priyamvada et al., 2021). It is not possible to cover all villages in the affected districts of the affected states using any ACD method, particularly the more intensive ones such as house-to-house searches, since Bihar alone has more than 43,000 villages in 33 endemic districts. A targeting strategy based on reliable predictions of villages with future cases is therefore essential. Because VL cases cluster in time and space, villages with cases in recent years are known to be at risk for more cases in the near future (Courtenay et al., 2017; Bulstra et al., 2018; Chapman et al., 2020). However, development of specific targeting strategies requires a quantitative expression of this clustering. Two quantitative components are combined to produce the strategy, the village-level case threshold that triggers ACD and the period of time over which these cases are counted. This article explores a range of case thresholds and time windows within KAMIS data from 2013 to 2019 to refine our strategy for village-level targeting of ACD and other public health interventions.

## Methods

As implemented in India since 2017, ACD is launched in a village as soon as a new VL case is confirmed. ACD includes two major search mechanisms: 1) case identification based on the index case’s knowledge of other known VL cases and searches in nearby houses (snowballing); and 2) sustained contact over time with a range of local informants and private providers, both formal and informal. Contacts with key village informants, for example, community level health workers, occur fortnightly throughout the period of ACD, with the aim of detecting cases early in the course of illness.

The initial village targeting strategy was derived from observations related to the indoor residual insecticide spraying (IRS) program for VL, which is closely supported by CARE India. The list of villages targeted for a given round of IRS comprises villages that have reported at least one VL case in the previous three years. In 2017, the IRS list contained over 8,500 villages, which was not a practical target for ACD. It was observed that these villages accounted for around 75% of the subsequent year’s cases, but the largest proportion came from villages affected in the preceding one year. To provide the best balance of feasibility and expected yield, the implemented ACD targeting strategy focused on villages with at least one VL case in the previous year. The current analysis reviews VL surveillance data generated since 2013 to evaluate the full range of options for targeting ACD.

### Data Source

The current analysis utilizes surveillance data from KAMIS, which maintains a live line list of VL and PKDL cases reported since 2013. Line listings are available for download by external users from the KAMIS application only as deidentified data. Personal identifiers are held confidentially in a limited-access, password-protected database. While the case lists are incomplete for earlier years, they are increasingly accurate for later years, with each case having been traced to their home and verified several times over the years. In addition, all village names in each affected block, as in use by the block health authorities, are included in the master list of villages in the online application, based on ground-verified names, locations, and estimated populations. Village center geolocation data are linked to each case. This permits accurate analysis of village-level case incidence patterns over time. The remaining two affected states, West Bengal and Uttar Pradesh, have used KAMIS in a similar manner since 2017 and 2019, respectively. In all, over 50,000 VL and PKDL cases are available in the database as of 2020.

The availability of accurately localized case data over nearly eight years permits detailed analysis of VL occurrence in time and space to advance understanding of disease transmission, improve predictions of the likely location of future cases, and improve targeting of ACD efforts. Such analyses, repeated several times over the last four years, have helped refine ACD strategies to monitor outcomes closely. The analysis presented here is based on line-list data downloaded from KAMIS on 12 October 2020. Since Bihar constitutes the large majority of reported VL cases in the country and since completeness and quality of data are more consistent over time, the current analysis is limited to Bihar. However, the patterns appear to be similar for the four affected districts of Jharkhand as well. The main variables from the KAMIS case line-list used for these analyses were village, type of disease (VL or PKDL), and date of diagnosis.

### Statistical Analyses

Our analyses tested past patterns of VL incidence at the village level as predictors of future incidence. We examined the risk of additional cases in villages reporting any case compared to those with no cases reported in previous years and tested the impact of using differing past time windows to define affected villages. To assess the kinetics of case appearance, we also computed the time in days between diagnosis of one VL case in a village and that of the next VL case in that same village and graphed the median times to the next case across all affected villages for every consecutive pair of cases (for instance, first to second case, second to third, and so on). We examined these patterns in parallel with the village-level risk of additional VL cases across the range of cumulative village case-loads. Finally, we analyzed the trade-off between ACD effort (number of targeted villages) and yield (new cases detected), as it varied by village-level cumulative case-loads using different past time windows (for instance, cases cumulated across past 3 *versus* past 5 years). Data were analyzed using Excel version 16.0, Python version 3.6.7, Pandas version 0.25.3, and NumPy version 1.17.4.

## Results

The KAMIS master list includes 43,880 villages in Bihar, of which 10,494 (23.5%) reported VL cases between 2013 and 2019. In addition, 314 villages reported cases of PKDL but no cases of VL. Thus, in total, 10,808 (24.6%) villages were ‘ever affected’ in this time window. Although the number ‘ever affected’ will increase each year, the growth is likely to be very slow as long as current low VL incidence continues: as of October 2020, only 181 ‘new’ villages had been added in 2020, barely 0.4% of the state denominator. Nevertheless, this tiny number accounts for 17% of all cases in 2020.

**Figure 1:**
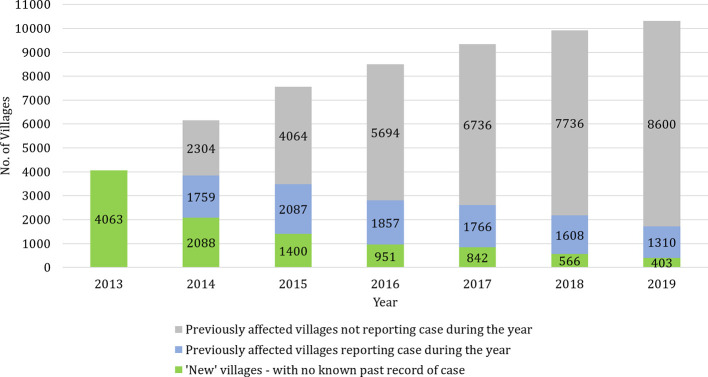
Occurrence of visceral leishmaniasis cases in previously affected and unaffected villages each year. The stacked bars show the number of ‘new’ villages and ‘previously affected’ villages with and without cases from 2013 to 2019. Since no village-level VL case data were available prior to 2013, all affected villages that year are considered to be ‘new’.

**Figure 1** shows the breakdown of cases in KAMIS by ‘new’ villages with cases, and ‘previously affected’ villages with and without cases each year. Since no village-level VL case data were available prior to 2013, all affected villages that year are considered to be ‘new’. As the case incidence fell over this time period, the proportion of villages with cases in a given year decreased. In 2013, the year with the highest annual caseload, 9.3% (4063/43,880) of villages were affected, and this proportion shrank to 3.9% in 2019 and to 2.1% in 2020 up to October. The proportion of ‘new’ villages among each year’s affected villages decreased from 54.3% in 2014 to 23.5% in 2019. At the same time, the proportion of ‘previously affected’ villages without cases rose steadily each year over the time period. In 2019, 8600/10494 (82%) of known ‘previously affected’ villages did not report any cases.

**Figure 2:**
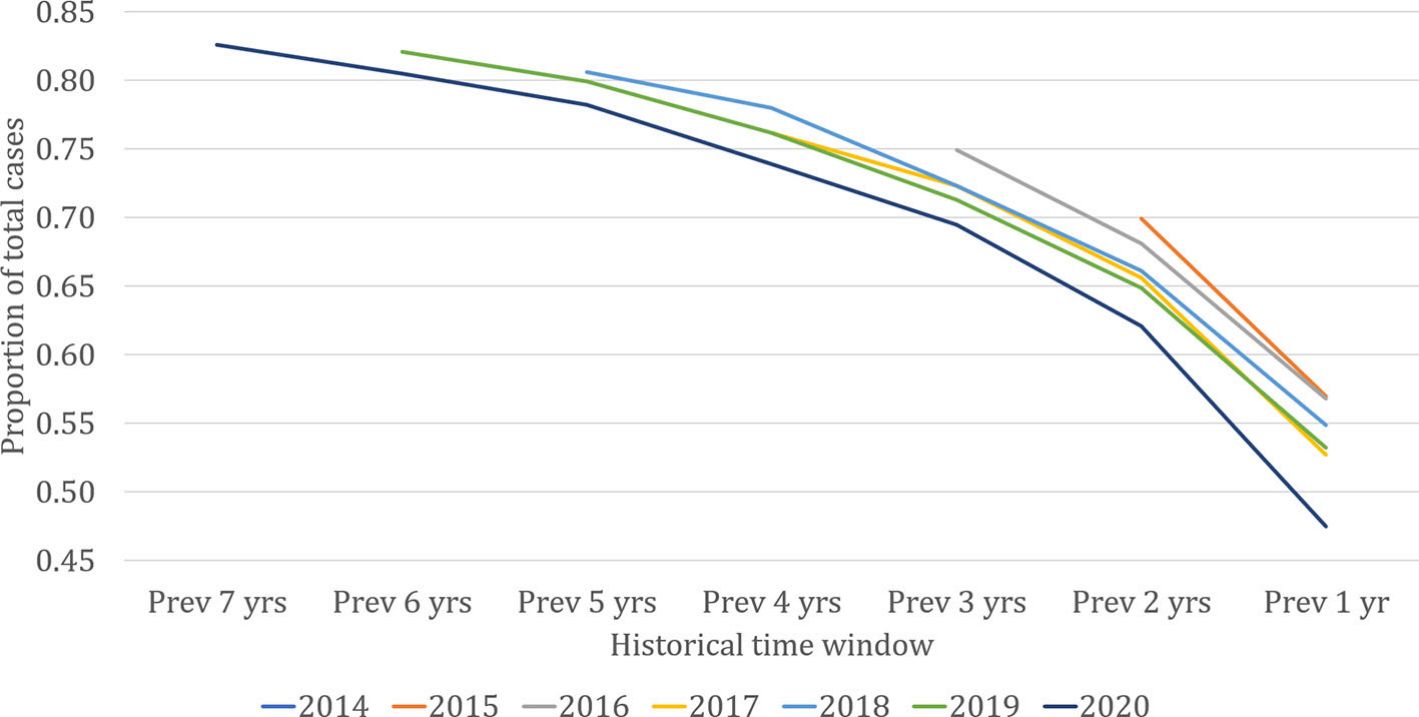
Proportion of visceral leishmaniasis cases each year coming from previously affected villages, 2013 to 2020 (up to September). The lines represent the cumulative proportion of all cases diagnosed in each of the last seven years that came from villages with cases in the historical time window on the horizontal axis.

From the vantage point of the present, past village-level data may provide predictive power for where cases are likely to occur in the near future. **Figure 2** shows the proportion of VL cases in each year that came from ‘previously affected’ villages. The lines represent the cumulative proportion of all cases diagnosed in each of the last seven years that came from villages with cases in the historical time windows on the horizontal axis. The proportion of each year’s cases coming from previously affected villages increased with increasing length of the historical time window. Approximately 55% of cases each year came from villages with cases reported in the previous year, around 65% from villages with cases in either or both of the previous two years, and so on. The remaining cases came from ‘new’ villages with no record of past cases in KAMIS. For the three longest time windows (5, 6, and 7 years), the proportion of cases coming from ‘new’ villages was consistently less than 20%, suggesting that in the timescales that the cumulated KAMIS data represents, the contribution to current cases of villages affected any longer than five years ago is likely to remain small. Given the limitation of available data, it is not clear what proportion comes from villages truly unaffected in the past.

**Figure 3:**
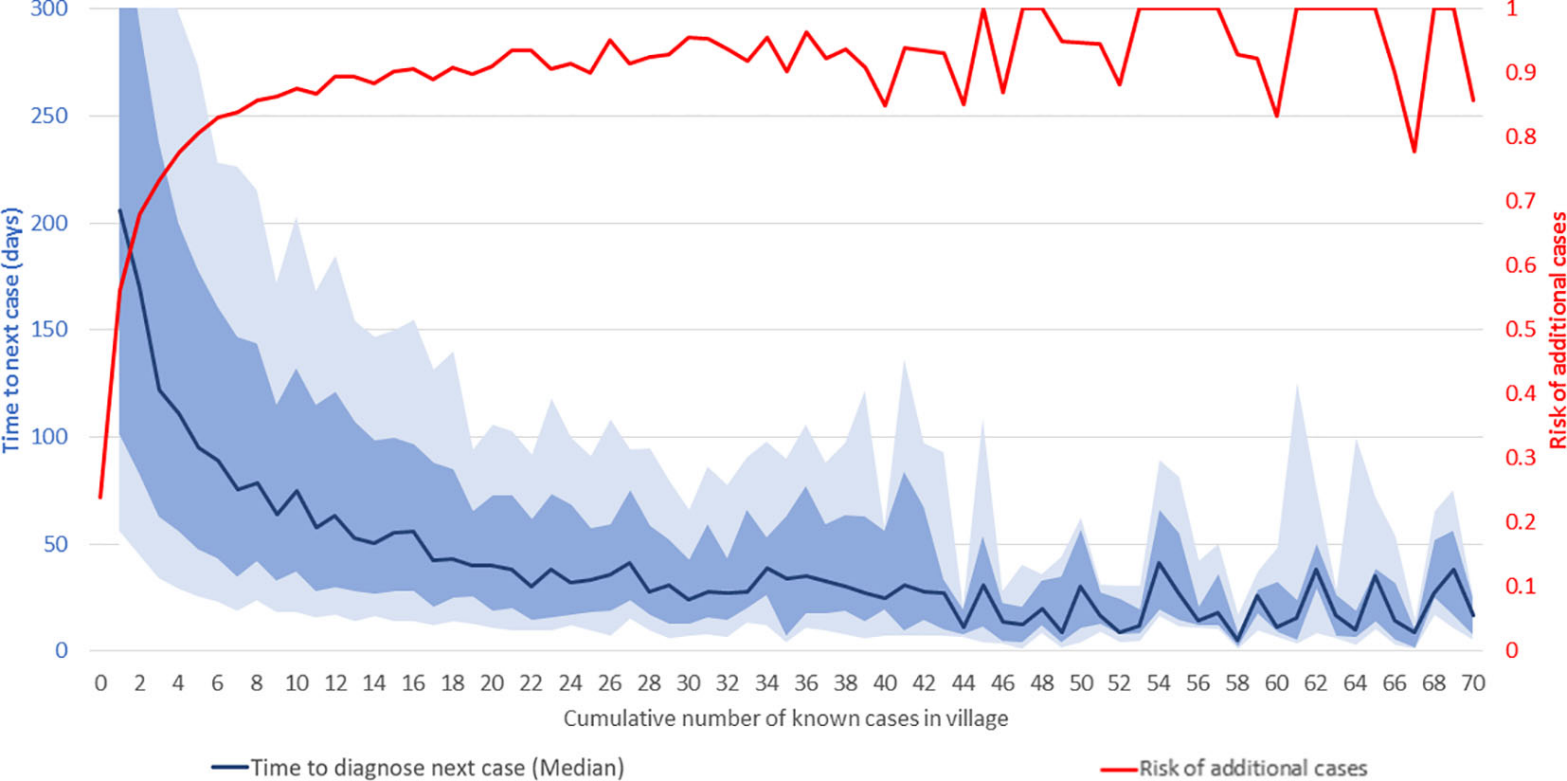
Time-to-next-case and risk of additional visceral leishmaniasis (VL) cases in affected villages. The horizontal axis shows the cumulative number of known VL cases at the village-level from January 2013 to September 2020. The red line shows the risk of additional VL cases (scale on right-hand vertical axis). The dark blue line shows the median interval in days to the next VL diagnosis in the village (scale on left-hand vertical axis). The light and medium blue shading indicates the ranges from the 25^th^ to 75^th^ percentiles and 35^th^ to 65^th^ percentiles, respectively. The horizontal axis is truncated at 70 cases and the left-hand vertical axis at 300 days.

At the village level, the risk of additional VL cases beyond the first case, and the time interval to their occurrence, vary with the cumulative case-load. **Figure 3** shows the median time in days from one VL diagnosis to the next (dark blue line) and risk of additional cases in a given village (red line) as functions of the cumulative number of known cases in that village throughout the study period. As shown on the right-hand vertical axis, 23.9% of villages reported one or more cases during this period. Of these villages, 56% had at least one more case, and 68% of those, in turn, had a third case or more, and so on. The risk of additional cases, indicated by the red line, increases steeply with increasing cumulative incidence in the village, reaching approximately 90% in villages with 12 cases and 100% in villages with 45 cases. Above 45 cases, additional cases are virtually inevitable, and the level of risk effectively remains at 100%. Clearly, villages with high cumulative case-loads should be targeted for ACD as they are virtually guaranteed to yield more cases. However, the vast majority of villages at risk have case-loads of 1–15, and there is considerable variation in risk of additional cases for villages in this range. Decisions about adding villages to be targeted from this range of cumulative case-loads must balance expected yield with attendant effort and available resources.

As shown by the dark blue line, the increasing risk of additional cases with increasing cumulative case-loads manifests as a progressively decreasing interval between the diagnoses of consecutive cases. Given the limitations of data availability in earlier years, the true ‘index’ case in a village cannot be identified with certainty. However, after the first reported case, the median time to the second case was 206 days, from the second to the third 170 days, and from the third to the fourth 122 days, and so on. The decline in the median time to the next case slows down as the cumulative number of cases in the village increases, and it reaches 30 days after about 30 cases. As might be expected, given variations in transmission, incubation periods and diagnosis effort, the variation around the median is large, as shown by the inter-percentile ranges in light (25^th^ to 75^th^) and medium (35^th^ to 65^th^) blue shading. For instance, the median time from the second to the third case was 122 days, with 25^th^, 35^th^, 65^th^, and 75^th^ percentiles of 34, 63, 238, and 342 days, respectively.

Based on these analyses, decisions on targeting of villages for ACD can consider the trade-off between effort (number of villages targeted) and VL case yield. As seen in **Figure 2**, 82% of the cases of 2019 came from villages that reported cases in the 6-year time window from 2013 to 2018. However, capturing these cases through ACD would have required targeting all 9,910 villages known to have had cases up to the end of 2018. As shown in **Figure 3**, more severely affected villages produce more subsequent VL cases and produce them more rapidly, but this relationship is steepest at the lower end of the case-load, with very little further increase in risk past around 15 cases.

**Figure 4:**
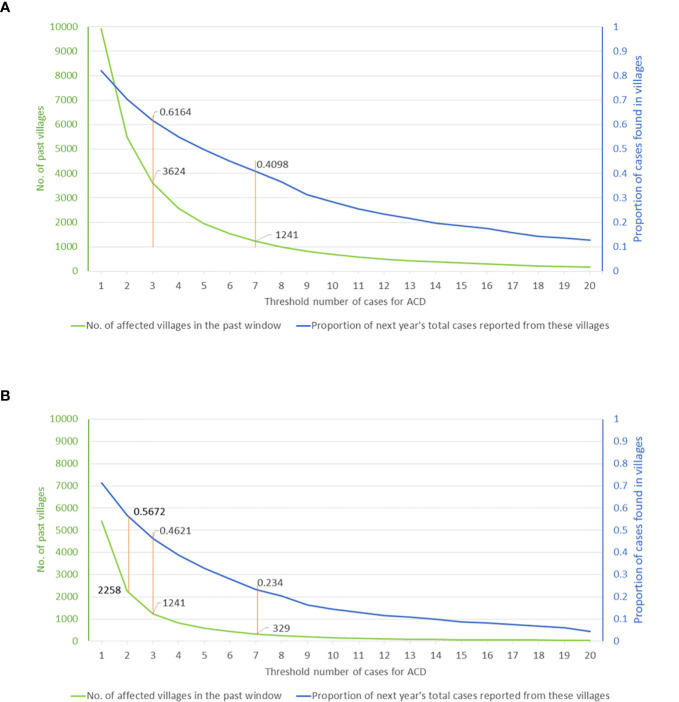
Visceral leishmaniasis case yield in 2019 from village-level targeting of active case detection activities **(A)** based on cumulative case-load during a 6-year window (2013–2018) or **(B)** the most recent 3-year window (2016–2018). The graphs can be used to assess the operational impact of selecting different thresholds of cumulative village case-load from the chosen time window (horizontal axis). The potential yield, defined by the proportion of the cases of 2019 potentially detected, is shown by the blue curve (right-hand vertical axis). The number of targeted villages is shown by the green curve (left-hand vertical axis).

Graphing the relationship between ACD effort and the resulting yield thus provides a tool for programmatic decision making. Based on cumulative village case-loads for 2013–2018 and the range of targeting thresholds, **Figure 4** shows the potential yield as a proportion of the cases of 2019, indicated by the blue curve. The effort, expressed as the number of targeted villages, is shown by the green curve. Using the full 6-year time window (2013–2018), a threshold of seven (or more) cases during this period selects 1,241 villages and potentially yields approximately 41% of the cases of 2019, whereas a threshold of three (or more) cases corresponds to 3,624 villages and a potential yield of 62% of the cases of 2019 (**Figure 4A**). If the past time-window is shortened, the overall yield of cases is lower for a given threshold, although the number of cases per targeted village is higher. For instance, using a 3-year window (2016–2018), a threshold of seven VL cases selects 329 villages and yields 23% of the cases of 2019, while a threshold of three cases selects 1,241 villages and yields 46% of cases of 2019 (**Figure 4B**). Thus, in 2019, targeting villages having three or more cases during the previous three years would have yielded twice as many cases as targeting villages having seven or more cases, but would have required nearly four times the effort (number of villages where ACD will have to be implemented). Understanding the nature of this trade-off will be crucial to operational decisions about deployment of limited resources.

## Discussion

India has now achieved lower annual VL case-loads than at any time in the past five decades (Sanyal et al., 1979; Sanyal, 1985; Rijal et al., 2019). A precise assessment of how close the subcontinent came to interruption of VL transmission during the blanket DDT spray campaigns for malaria elimination in 1955–1964 is impossible due to the lack of systematic VL surveillance during that time period (Sanyal et al., 1979; Sanyal, 1985). However, *Leishmania donovani* genetic data show a tight bottleneck at the corresponding time period suggesting elimination of a large proportion of the parasite population in the Indian subcontinent (Downing et al., 2011; Imamura et al., 2016). Nevertheless, a resurgence of VL transmission was already evident to informed observers by the mid-1970s and had increased to more than 60,000 reported cases in 1978 (Sen Gupta, 1975; Sanyal, 1985). Undetected cases of post-kala-azar dermal leishmaniasis (PKDL) have been suggested as a major interepidemic reservoir (Addy and Nandy, 1992). Two subsequent VL epidemic cycles, with peaks in 1992 and 2007, followed that resurgence (Courtenay et al., 2017). To ensure that any future resurgence is detected early, a sustainable plan for VL surveillance and early case detection is crucial. An estimated 90% of PKDL cases occurs after apparently successful treatment of VL (Islam et al., 2013); thus the most efficient ACD method for PKDL consists of systematic follow-up of treated VL patients (Dubey et al., in press).

Our analyses have limitations related to the nature of routinely collected surveillance data. The major limitation is the lack of data at the local level prior to 2013. A large proportion of the villages defined as “newly affected” in 2013 were almost certainly previously affected; we can only analyze the patterns for the downward slope of the most recent epidemic cycle. A related limitation is the incompleteness of line list data from earlier years in the KAMIS database, particularly prior to 2017; thus, the interpretation of patterns as presented should not be read as being highly precise, but as being closely indicative. The other fundamental element enabling such analysis, which required a huge effort over several years, has been the establishment of a verified master-list of villages categorized by health subcenter and block within KAMIS. Once this was in place, and once every case had been accurately mapped to his or her resident village in the master-list, village-level analysis of case patterns over the entire endemic zone of Bihar became possible for the first time.

Our analyses demonstrate that the location of the majority of future cases is predictable and that the most recently affected villages are most vulnerable to further case incidence. However, ‘new’ villages appear each year and will continue to surprise the elimination program unless other methods of prediction, for example spatiotemporal modeling (Mandal et al., 2018; Nightingale et al., 2020), prove effective and applicable at a program level. The finding that risk increases and the time to the next case decreases with every additional case in a village is revealing, but epidemiologically intuitive. Higher case density is expected to lead to increased transmission in a vicious cycle. These findings highlight the value of examining cumulative case-loads over prolonged periods. However, as with all augmentation of data for prediction, there is a trade-off between the effort required and maximizing yield per unit effort (in this case, villages targeted for ACD). Explicit analyses of these trade-offs, which might change as VL case-loads and incidence patterns change, can guide programmatic decisions on investment in ACD and other interventions.

The observed patterns of “time-to-next-case” provide a potential evidence-based approach to defining outbreaks. Conceptually, cases occurring faster than expected in a given community constitute an outbreak (Murhekar et al., 2009). In the current analyses, the time to each additional case diminished with increasing cumulative case-load, suggesting that an outbreak definition of a fixed number of cases occurring per unit of time may be inappropriate. Graphs of cumulative case-load and timing, as in **Figure 3**, could be made interactive, such that when the interval to diagnosis of a new case in a village falls some distance below the median line, it would be an indication that the new case has appeared faster than expected and might be the harbinger of more cases. In the context of a live electronic database such as KAMIS, an algorithm could be implemented to alert the appropriate users instantaneously when a newly reported case triggers a potential outbreak alert. Given the many small and large outbreaks in the eight-year time window of KAMIS data, an appropriate threshold could be empirically derived and then prospectively tested. To date, initiation of outbreak investigations has required reporting of case clusters by astute local staff (Priyamvada et al., 2021).

Another application of time-to-next-case curves could be the evaluation of different interventions to decrease VL transmission. Effective vector control should potentially move the median to the right, prolonging the time to the next case by reducing efficiency of transmission. Interestingly, ACD, where the primary purpose is to reduce time to diagnosis, should move the median downwards (to the left) in the immediate period, but in the long run will lengthen the median time-to-next-case, as the shortened interval that VL patients remain untreated and therefore infectious translates into decreased transmission in that community.

In conclusion, the foregoing analysis suggests the efficiency of ACD can be optimized by prioritizing recently affected villages and those with higher cumulative case-loads. As innovations are made in ACD methods, these can be incorporated into the surveillance system (Khatun et al., 2014). While it is impossible to predict the location of every future case in advance, it is clear from the analyses presented that it is possible to predict the location of a large proportion of future cases (around 82% for 2019, for instance). The trade-off between ACD effort (numbers of villages targeted) and yield (in additional cases detected) can be computed to provide an accurate estimate of cost and efficiency. Such detailed data over the range of options can provide decision-makers with the information to choose a balance of investment, in human resource and economic terms, *versus* the projected level of case detection through such effort. In practice, the decision will depend upon the operational efficiencies of existing programs and the feasibility of implementation of a given ACD approach. The maintenance of a sufficiently sensitive, comprehensive VL surveillance system into the future will be crucial for the early detection of case clusters and potential VL resurgence.

## Data Availability Statement

The raw data supporting the conclusions of this article will be made available by the authors, without undue reservation.

## Ethics Statement

The study was and approved by the Ashirwad Ethics Committee, Ashirwad Hospital and Research Center, Ulhasnagar, Maharashtra, India. Written informed consent for participation was not required for this study in accordance with the national legislation and the institutional requirements.

## Author Contributions

JB, KP, LACC, CB, and SS developed the concepts and designed the study methods. JB, BR, IB, and BS established and managed the data collection system. JB, LACC, CB, AH, and SS analyzed the data. JB, LACC, CB, and SS prepared the figures, interpreted the results, and wrote the manuscript. KP, TM, BR, IB, BS, AH, MS, and ND reviewed and commented on the manuscript. All authors contributed to the article and approved the submitted version.

## Funding

This study was financially supported by a grant from the Bill and Melinda Gates Foundation (Grant ID# OPP1196454), http://www.gatesfoundation.org/. The funder had no role in study design, data collection and analysis, decision to publish, or preparation of the manuscript. LACC was supported by the Bill and Melinda Gates Foundation through the Setting the Post-Elimination Agenda for Kala-azar in India consortium (Grant OPP1183986).

## Conflict of Interest

The authors declare that the research was conducted in the absence of any commercial or financial relationships that could be construed as a potential conflict of interest.
